# Psychological Science within a Three-Dimensional Ontology

**DOI:** 10.1007/s12124-017-9412-8

**Published:** 2017-12-12

**Authors:** Lars-Gunnar Lundh

**Affiliations:** Department of Psychology, Lund University, Lund, Sweden

**Keywords:** Ontology, Psychological science, Materialism, Emergentism, Phenomenology, Social constructionism

## Abstract

The present paper outlines the nature of a three-dimensional ontology and the place of psychological science within this ontology, in a way that is partly similar to and partly different from that of Pérez-Álvarez. The first dimension is *the material realities,* and involves different levels (physical, chemical, biological, psychological, etc.), where each level builds on a lower level but also involves the development of new emergent properties, in accordance with Bunge’s emergent materialism. Each level involves systems, with components, structures and mechanisms, and an environment. This dimension can be studied with natural scientific methods. The second dimension is *the subjective-experiential realities*, and refers to our subjective perspective on the world. In accordance with Husserl’s phenomenology, it is argued that this subjectivity does not exist *in* the world (i.e., should not be reified as an object among other objects), but represents a perspective on the world that we enter in our capacity as conscious human beings. Essential characteristics of this subjectivity (such as intentionality, temporality, embodiment, and intersubjectivity) can be explored by phenomenological methods. The third dimension is *the social-constructional realities*, and includes social institutions, norms, categories, theories, and techniques. It is argued that psychological science spans over all three dimensions. Although almost all psychological research by necessity starts from a problem formulation where the subjective-experiential dimension plays an essential role (either explicitly or implicitly), most of present-day psychological research clearly emphasizes the material dimension. It is argued that a mature psychological science needs to integrate all three dimensions.

In his article “Psychology as a science of subject and comportment, beyond the mind and behavior”, Marino Pérez-Álvarez (2017) argues for a reconceptualization of psychological science within a three-dimensional ontology. This is the starting-point for the present paper. How can we understand psychological science within a three-dimensional ontology, and how are these three dimensions to be defined? The paper has five parts. In the first part, it is argued that ontological questions are essential to the development of psychological science. In the three following sections, the ontological dimensions are outlined. The fifth and final part contains a discussion of some similarities and differences between the present version of a three-dimensional ontology and that of Pérez-Álvarez.

## Ontology and Psychology

Ontology is that part of philosophy which deals with questions about the nature of what *exists*, and how different aspects of being are related to each other. Examples of ontological questions directly relevant to psychology are: What is the nature of consciousness, sensation, perception, memory, thinking, feeling, intention, expectation, will, desire, hope, fear, joy, sadness, love, anger, empathy, etc.? Or, to turn to more theoretical concepts: What is the nature of human information processing, personality, personal identity, interpersonal relations, etc.? And how are all these things related to each other, and to the non-psychological (physical, chemical, biological, social, etc.) world? Traditional questions in this area are formulated in terms of how the “mind” is related to (1) external reality, (2) the body, (3) the brain, (4) language, and (5) human society and culture.

One classical philosophical position, often traced to Descartes, is that of mind-body dualism. According to dualistic thinking, mind and body represent different kinds of realities (“substances”, in Descartes’ thinking). Pérez-Álvarez (2017) argues forcibly against dualism, and in favor of *pluralism*. A pluralistic ontology, he says, “does not reduce reality to two substances (dualism) or to one (monism). Realities have many forms: experiential (pain, feelings, thoughts), physical (electrons, atoms, cells, organisms, typewriters, planets), institutional (languages, cultures, family relationship systems, collective imaginaries, world views) and abstract (mathematics, theorems, theories, geometry).”

The position in favor of pluralism that is first taken by Pérez-Álvarez (2017) is later qualified in at least two different ways: (1) first by abandoning “strong pluralism” in favor of a “tripartite” ontology, which implies a reduction of a potentially pluralistic universe into three different “worlds”, and then (2) by qualifying this tripartite ontology by a streak of monism, in the sense that one of these “three worlds” (i.e., physical reality) is seen as more basic than the others (materialism). In both of these aspects he follows Bueno’s philosophical materialism, which involves three “genres of materiality”: (1) the physical world, including all kinds of material bodies from electrons to planets (M1); (2) the subject with its subjective experiences and behavioral activity (M2); and (3) abstract entities, such as concepts, mathematics, social institutions, and cultural things (M3).

Basically, I find the notion of a three-dimensional otology appealing, although don’t want to call it “tripartite”, because this suggests the idea of three different “parts” of reality that are somehow disconnected. The notion of “three-dimensional” rather suggests an analogy with three-dimensional space, in which three parameters (i.e., width, height, and depth) are required to determine the position of any element. In this paper I will discuss such a three-dimensional ontology in slightly different terms than Pérez-Álvarez, by labeling the three dimensions *material*, *subjective-experiential*, and *social-constructional*. Also, rather than referring to the three dimensions as different “worlds”, I will choose a more “pluralistic” discourse by referring to them as three different *realities*. Because each of these three dimensions can be assumed to be characterized by a certain degree of ontological pluralism, I will use “realities” in plural also to characterize each dimension.

This also means that I don’t subscribe to seeing these three dimensions as “genres of materiality”, or identifying the subject of psychology specifically with the second dimension (as Pérez-Álvarez (2017) does). In the following, I will argue (1) that only the first dimension (the material realities) is strictly material, and (2) that psychological science cannot be restricted to only one of these dimensions but spans over all three of them.

## Dimension 1: The Material Realities

This ontological dimension refers to the realities that can be studied by the natural sciences, and could also be referred to as the “physical-natural” dimension. In the following exposition, this dimension is approached from the perspective of Bunge’s (1977, 2010) emergent materialism.

### Emergent Materialism

In this model, the material realities are differentiated into physical, chemical, biological, psychological, and sociological levels, where each level builds on a lower level but also involves the development of new emergent properties. Simply put: Physics involves the level of atoms. Chemistry involves the study of molecules, which are composed of atoms but have emergent properties that atoms do not have. Biology in turn involves the study of cells, which are composed of molecules, but yet have new emergent properties that are not found in molecules, etc.

Two basic assumptions in Bunge’s emergent materialism are that (1) each system has emergent properties that are not found in its components, and yet (2) “each emergent property of a system can be explained in terms of properties of its components and of the couplings amongst these” (Bunge 1977, s. 503). In other words: even if the emergent properties of a system are new in the sense that the components of this system do not have these properties, it is nevertheless the case that these emergent properties can be *explained* in terms of properties of these components and the relations between these. Methodologically, this means that each system as a rule should be studied at two levels, both a macro level and a micro level (i.e., the level of the components).

To illustrate: At their macro level physical objects have a number of emergent properties, such as temperature and entropy, which their components at the micro level (e.g., molecules, atoms, elementary particles) do not have. Molecules, for example, do not have temperature, and yet the temperature of physical objects can be *explained* in terms of properties of the molecules that they consist of. The temperature of a physical object depends on how much the molecules in this object are moving; the more they move, the higher the temperature.

Important here is that no new “substance” is added at any of these levels of material reality. For example, although living matter has a number of emergent properties, such as cellularity, metabolism, homeostasis, cell division, heredity, mutation, morphogenesis, evolution, sickness, and death, all of these are possible to explain in terms of the material building bricks that are involved. The same is, according to Bunge’s *psychoneural identity theory*, true of psychological processes (such as thought, emotions, conscious awareness and free will), which are seen as emergent properties of “psycho-systems” that are based on processes in the central nervous system of living organisms.

Among these emergent properties are self-control and free will. Bunge describes evolution as being “in part a liberation process: one of decreasing dependence on the environment (thanks to improvements in homeostasis) and increasing empowerment (ability to do), thanks to improvements of the brain and its social uses” (Bunge 2010, p. 220). This increasing independence is seen not only in the fact that the brain is continuously active, even during sleep, and that “most brain processes are spontaneous or self-generated rather than responses to external stimuli” (p. 220), but also in the fact that “self-control, which is a necessary condition for free will, is a learnable function of the prefrontal cortex, the phylogenetically newest area of the brain. So much so, that people with serious damage to that brain region lack free will: they are swayed by the stimuli impinging on them” (p. 220).

According to Bunge, an important implication of emergentism is that psychological processes by necessity rely on biological processes, which makes it impossible for computers to develop consciousness, feelings, and free will. The mental functions, according to this model, cannot be decoupled from the living body which they are part of.

Another important part of Bunge’s emergent materialism is that each system involves not only *components*, but also an internal *structure*, internal *mechanisms* and an *environment* (Bunge and Ardila 1987). This means that the system cannot be defined apart from its environment. Each system has its specific kind of environment, the nature of which depends on the properties of the system in question. An interesting implication of this is that environments also exist at different levels. To take an example: Water is an essential part of our environment, without which life would not be possible. A common reductionistic mistake is to assume that water can be reduced to the chemical molecule H_2_O. But water has emergent properties, that are essential to life, but are not found in the chemical H_2_O molecule:

To state that the *composition* of a body of water is a set of H_2_O molecules is not to state that the former is nothing but the latter… a body of water is a system, hence something with a structure, not only a composition. And that structure includes the hydrogen bonds among H_2_O molecules. The result is a system with emergent properties such as fluidity, viscosity, transparency and others, which its molecular components lack (Bunge 1977, p. 506).

These emergent properties are what makes water” drink-able”. This reasoning is consistent with biological notions of an “Umwelt” (Von Uexküll 1957) and Gibson’s (1979) theory of affordances, which also point to important holistic aspects of organism-environment systems.

### Functions, Not Experiences

An important limitation is that Bunge’s emergent materialism stays strictly within the material dimension, and has nothing to say about what Chalmers (1995) calls “the hard problem of consciousness”. This is seen, for example, in Bunge’s way of formulating empirically testable hypotheses derived from his theory in *functional* terms. As he formulates it: From the assumption that “for every mental function F there is a brain system B that performs F” it follows that, “*If B is injured or absent, F is disturbed or fails to occur”* (Bunge 2010, p. 162). This is an empirically testable hypothesis because “it is possible to alter neural systems through pharmacological or surgical means, or else TMS (transcranial magnetic stimulation), and measure the resulting changes in behavior” (Bunge 2010, s. 163). More generally, research on neural plasticity shows that the brain undergoes neurophysiologically measurable changes as the result of new learning (e.g., in connection with language learning or successful psychotherapy).

According to Bunge, consciousness belongs to the emergent properties of psycho-systems. Chalmers (1995), however, argues that consciousness is an ambiguous term, which covers a number of different problems, of which some are “easier” and others are more difficult. If by “consciousness” we merely mean merely the ability to report about one’s inner mental states, it would be possible to define it in terms of a mechanism that performs this function, and would therefore fit neatly into Bunge’s emergentism. What Chalmers is primarily interested in, however, is not this relatively “easy” problem but the considerably more difficult problem of explaining how and why we have conscious, subjective *experiences*. Easy problems, he argues, can be solved relatively easy because all that they require is *a mechanism that can perform a particular function*. The hard problem of conscious experience, on the other hand, will persist even when the performance of all relevant functions is explained.

### Levels are Not Dimensions

It is important not to mix the *levels* in emergent materialism with ontological *dimensions*. These levels (whether physical, chemical, biological, psychological or social) are all part of the material dimension. Chalmers’ “hard problem of consciousness”, however, is not a problem of how psycho-systems are related to lower-level systems, but a problem of how the subjective-experiential dimension is related to the material dimension.

This is an important distinction. For, although it is in principle possible to find cognitive or neural mechanisms that perform all kinds *of information processing*, this still does not explain the conscious *experience* that is involved.

The really hard problem of consciousness is the problem of *experience*. When we think and perceive, there is a whir of information-processing, but there is also a subjective aspect. As Nagel (1974) has put it, there is *something it is like* to be a conscious organism. This subjective aspect is experience. When we see, for example, we *experience* visual sensations: the felt quality of redness, the experience of dark and light, the quality of depth in a visual field. Other experiences go along with perception in different modalities: the sound of a clarinet, the smell of mothballs. Then there are bodily sensations, from pains to orgasms; mental images that are conjured up internally; the felt quality of emotion, and the experience of a stream of conscious thought. What unites all of these states is that there is something it is like to be in them. All of them are states of experience. (Chalmers 1995, p. 201)

As Chalmers points out, what makes the hard problem of conscious experience difficult is that it cannot be reduced to problems about the performance of functions. The problem is to explain why the performance of certain cognitive functions is accompanied by *subjective experiences*. He refers to this as “an explanatory gap”, and says that we need an “explanatory bridge” to cross it: “A mere account of the functions stays on one side of the gap, so the materials for the bridge must be found elsewhere” (Chalmers 1995, p. 203). His suggested solution is to see conscious experience as a fundamental non-reducible property of the world, at the same level as other fundamental non-reducible entities such as mass, charge, and space-time. This leads him to a speculative double-aspect theory, similar to that of the seventeenth century philosopher Spinoza, with the implication that everything has an experiential aspect.

According to the three-dimensional ontology that is argued for in the present paper, Chalmers’ mistake is that he *objectifies subjective experience*. An alternative to this is to see subjective experience in terms of Husserl’s phenomenology, which offers another kind of ontological perspective.

## Dimension 2: The Subjective-Experiential Realities

This dimension refers to those realities that are subjectively experienced, and can be made a focus of study by turning our attention to conscious experience as such, while putting the external world “within brackets”. This is the task that Husserl (1938/1970) set for the science of phenomenology, which he regarded as essential for laying the conceptual framework of a psychological science.

The basic starting-point for Husserl’s phenomenology is that all objective knowledge about the world by necessity involves a subjective perspective on these objective realities. There is no way to access any objective realities without going via our subjective perspective – yet, we seldom focus on this subjective perspective as such. The phenomenological method is based on *a change in perspective*, so that attention is now turned directly to our subjective experience, while disregarding the realities of the external world – this change of perspective is referred to by Husserl as an *epoché*.

Setting his phenomenology into a historical context, Husserl (1938/1970) differentiates between the natural attitude, the theoretical attitude, and the phenomenological attitude. It is our *natural attitude* to engage in the world around us for practical everyday purposes, without focusing directly on our subjective perspective as such, but treating it rather as a kind of transparent medium for our access to the objective world. This has been equally true of the natural sciences. Although the development of science from ancient Greece onwards, as Husserl describes it, has involved the development of a *theoretical attitude,* in addition to our original natural attitude, this has seldom involved any attention given to our subjective perspective, but has mostly involved a theoretical refinement of the natural attitude into a *naturalistic attitude* (which reduces all of reality to the material dimension).

Although Husserl expressed great admiration for the developments that had occurred in the natural sciences, he emphasized that this progress had been one-sided in the sense that theoretical reflection had been applied only to the *object* of these sciences, and had forgotten to focus on the subject without which no objective knowledge would be possible. The phenomenological method is needed to “do justice to the subjectivity which accomplishes science” (Husserl 1938/1970, s. 295), and involves the taking of a *phenomenological attitude*.

### The Phenomenological Attitude

The phenomenological method is based on a change in perspective (an *epoché*), so that attention is now turned directly to our subjective experience. Husserl points out that this leaves everything just as it is (i.e., the existence of the external world is in no way questioned). The only change is a change of perspective: “through the epoché a new way of experiencing, of thinking, of theorizing, is opened to the philosopher; here, situated *above* his own natural being and *above* the natural world, he loses nothing of their being and their objective truths” (Husserl 1938/1970, p. 78).

What Husserl describes here can be compared with a number of concepts from modern psychology, such as “decentering”, “self-distant perspective, “cognitive distancing”, “meta-cognitive awareness”, “cognitive defusion”, and “mindfulness”. Common to all of these concepts is that they refer to the “capacity to shift experiential perspective—from *within* one’s subjective experience *onto* that experience” (Bernstein et al. 2015, s. 599). One difference, as compared with Husserl’s epoché, is that these concepts have arisen within a psychotherapeutic context rather than a philosophical one and therefore emphasize the potentially therapeutic effects of acquiring this attitude, rather than its importance for philosophical or theoretical purposes. What is added specifically by Husserl’s phenomenology is that this phenomenological attitude to experience can be used for theoretic-analytic purposes.

It is important to remember here that our conscious experience represents our *view of* the world, and should not be reified as some kind of entity *in* the world, as is done by Chalmers (1995). The purpose is to analyze our subjectivity, but not as something which exists *in* the world (an object among other objects), but as essential characteristics of our subjective *perspective on* the world.^1^ In other words, the purpose is to study human subjectivity (conscious awareness) “from within”, by observing and analyzing the perspective on the world that we enter in our capacity as conscious human beings. Some of the most important of Husserl’s analyses focus on the following characteristics of our subjective perspective: intentionality, temporality, embodiment, and intersubjectivity. One aspect of these can be referred to as “indexicality”.

#### Indexicality

The subjective perspective can be defined in terms such as “I”, “here”, and “now”. These terms have been referred to as *indexical* terms (Peirce 1932), because they don’t point to specific physical places, moments and individuals, but to places, moments and individuals that vary depending on where, when and by whom the terms are used. In other words, these terms do not refer to objective physical or natural realities, but to a subjective *perspective* on these realities.

### Embodiment

The subjective perspective is closely connected with our body. As Husserl (1938/1970) formulates it, the living body is *a center for our experience of the world*, and an organ of the will. “In a quite unique way”, he says, “the living body is constantly in the perceptual field quite immediately, with a completely unique ontic meaning… as the ego of affection and action” (p. 107). One aspect of this is that the body is “the zero point” for our experience of time, space, orientation and movement, from which all directions (“here”, “there”, “up”, “down”, “left”, “right”, near”, “far”, etc.) get their sense. In other words, the position of our body determines our perceptual perspective on the world.

It is also part of our immediate experience that we can move our body, change position, actively explore the environment, and influence our surroundings in various ways, by an effort of will. Husserl (1938/1970, p. 217-218) speaks about this in terms of an experience of “holding sway” over the body, and of the body as “an organ of the will”. In addition to the experience that our body, like other physical objects, is subject to physical causality (e.g., gravitation), the experience of “holding sway” over the body, according to Husserl, indicates another kind of causality. Here it is important to bear in mind that this is an attempt to describe essential aspects of our subjective perspective, which is not the same as arguing that free will exists in a material sense. As emphasized by Husserl, the purpose of phenomenology is to describe experience, while putting the objective world “within brackets” – which means that questions about the physical or material status of free will are *not posed* here. (Still, these are real questions that need to be posed from the perspective of a three-dimensional ontology.)

### Temporality

Husserl regarded time as the most difficult phenomenological problem, and devoted much work to this topic. Basic to his analysis of temporality is a differentiation between (1) natural occurrences with a beginning, duration and an end, that can be located in the physical world, and (2) the temporal structure of our subjective experience, which cannot be reduced to a sequence of separate moments, but is “layered” in the sense that it essentially involves not only a *now moment*, but also *retention* (of previous moments) and *protention* (of anticipated moments). As emphasized by Husserl this does not involve memory or expectations in the strict sense, but rather implies that *perception takes place over time*. Consider, for example, our experience of a melody; if our subjective experience was simply a series of separate moments we might be able to experience one tone at a time, but we would not be able to experience any melody. What makes us able to experience the melody as a whole is that our consciousness is constructed in such a way that we are able to integrate temporal occurrences into larger unities.

This basic integration is seen by Husserl as the most fundamental form of *synthesis*. An example of a more complex form of temporal synthesis is the “ego synthesis”, in which the past, the present and the future are woven together into a historical unit that may cover a whole life-time (an “identity” in present-day terminology), and which shows a certain type of continuity in spite of change. Common to both of these kinds of syntheses, however, is that they are what Husserl (1931/2001) refers to as *passive* syntheses – as distinct from more *active* syntheses that involve conscious thinking and judgment.

This illustrates an important thing about Husserl’s phenomenology: to analyze subjective experience here goes far deeper than merely describing conscious experience – it involves analyzing the necessary conditions for the psychological phenomena involved.^2^ For example, without the ability to synthesize time (i.e., passively integrating the now moment with retention and protention), it would *not be possible* for us to perceive a melody, or to read and comprehend a complete sentence, or to follow an argument that proceeds over time. What is referred to by Husserl as “passive syntheses” (unlike active syntheses) are not represented in our subjective experience, but represent necessary conditions for it.

### Intentionality

Intentionality refers to the fact that most of our experiences (with some exceptions such as pain, which does not refer to anything else beyond the pain itself) are directed to some kind of “object” beyond the experience itself. Although this object may sometimes be quite imaginary (as, for example, when we think of a centaur), an important part of our engagement with the world around us is aimed at getting a correct apprehension of it. Husserl (1938/1970, p. 163) describes this in terms of our strivings for “ontic certainty” in the form of “a harmony in the total perception of the world”, which needs sometimes to be “sustained through correction”. This, again, refers to a kind of synthesis, although an active synthesis in this case, rather than a passive one, to the extent that we actively and consciously strive to correct our perceptions.

An alternative formulation that is used by Husserl in some of his writings is to make this contrast in terms of active versus passive *genesis.* This is specifically developed in his *genetic phenomenology*, where the purpose is to analyze the historical conditions for the development of a subjective perspective with its particular intentionality (e.g., Husserl 1931/2001).

### Intersubjectivity

Basic to our subjective perspective is that we perceive ourselves as one subject among other subjects – each with a separate body that defines a center of experience, with its subjective perspective on the world, and its specific intentionality. According to Husserl, we tend to understand the subjectivity of other individuals on analogy with our own. Just as our experience of the world takes it starting-point from the *here* (embodiment) and the *now* (temporality), our understanding of others’ subjectivity takes its starting-point from the *ego* (our own intentionality).

Our basic experience that there are other individuals around us, each with their specific subjectivity, leads to an experience of *intersubjectivity*. In our communication with these others, we learn to know some of them more than others, and through “reciprocal correction” we reconcile our subjective perspectives on the world, so as to arrive at some kind of intersubjective consensus. As a result, new syntheses are formed, from a simple “I-you synthesis” (based on connecting with another person on the basis of partly overlapping intentionalities) to “the more complicated we-synthesis” (Husserl 1938/1970, p. 172) which may involve larger groups of people with more or less similar interests and perspectives. More generally, Husserl concludes that in this way the world is intersubjectively constituted, as the result of a synthesis of individual intentionalities:

All the levels and strata through which the syntheses, intentionally overlapping as they are from subject to subject, are interwoven form a universal unity of synthesis; through it the objective universe comes to be… In this regard we speak of the “intersubjective constitution of the world”… out of elementary intentionalities. (Husserl 1938/1970, p. 168)

The intersubjective constitution of the world is important to the understanding of the scientific status of phenomenology as a method. When Husserl advocates a phenomenological analysis of how the world appears to us, he refers to something *we all have access to,* in our capacity as human beings. In other words, we are dealing with realities that are *intersubjectively available*. This makes it possible to conduct meaningful discussions of these analyses, where different arguments are advanced, criticized, corrected and developed in various ways. Even if this is not an empirical method in the traditional sense, it is nevertheless the case that conclusions on the basis of these analyses are provisional and hypothetical, and possible to test by means of new arguments based on experiences which we all have potential access to. This is important in view of the requirement for intersubjectivity in science – in order for knowledge to qualify for a scientific status it has to be the result of methods that make it possible for different researchers to replicate the same investigation and see if they get the same results.

Further, if the understanding that is arrived at through this intersubjective process achieves the status of socially accepted conceptualizations, it will have received the status of social constructions. This brings us to the third ontological dimension.

## Dimension 3: The Social-Constructional Realities

This ontological dimension refers to everything that is socially constructed by human beings, including social institutions, norms, art, literature, concepts, categories, theories, and techniques. The study of these things is a focus within various social sciences, cultural sciences, and human sciences. Here the social-constructional dimension will be discussed briefly under five subheadings: Social facts, norms, categories, theories, and techniques.

### Social Institutions

One of the early examples of a scientific focus on these kinds of sociocultural realities is found in Durkheim’s (1895/1982) definition of sociology as the empirical study of “social facts”, such as kinship and marriage, currency, language, religion, political organizations, and other social institutions that play a role in everyday interactions with other members of the society we live in. What characterizes such social facts, as distinct from natural facts, is that they have no separate existence apart from the individuals who take them as facts; in this sense they are *social constructions*. Still, they are there “objectively” as realities to explore by means of various methods.

In Searle’s (1995) terminology, such institutional social facts are “epistemologically objective” – that is, they are there for us to study and gain knowledge about – while at the same time being “ontologically subjective”. Searle’s use of the term “subjective”, however, is likely to mislead, and is not consistent with the present perspective where the term “subjective” is used specifically to refer to conscious experiences, which belong to the second ontological dimension. A better formulation might be that social constructions are ontologically *intersubjective* – that is, they require some degree of at least implicit intersubjective “agreement” for their existence (cf. Wittgenstein’s (1953) arguments against the possibility of a private language).

In general, the social reality includes a large variety of social constructions which, although they exist only by virtue of the fact that we treat them as real, affect our lives in a number of different ways. A classical expression of this in sociology is the Thomas theorem:” If people define situations as real, they are real in their consequences” (Thomas and Thomas 1928).

### Norms

What is true of social facts in general is also true of norms, defined as beliefs of what “ought” to be. That is, although norms have no separate existence apart from the individuals who take them as norms, they still are there as “objective” social-constructional realities to be explored by various methods. Norms can be more or less specific to various social groups – from the family and other small-scale groups, over scientific disciplines and religious groups, to entire societies and cultures. Norms may refer to what is seen as acceptable or unacceptable conduct, but they may also refer to ideals to strive for.

### Categories

All kinds of categorizations are social constructions, created by human beings for the purpose of bringing some kind of order in the multiplicity of things that exist. An important difference between categorizing human beings and categorizing physical matter, however, is that the former type of categorizations can affect the realities that they aim to describe in a way that the latter cannot. Hacking (1999) describes this by referring to the former as *interactive categories*. To illustrate: Classifying certain entities in physics as “quarks” does not affect the quarks. Classifying an individual as a “man” or a “woman”, however, affects both the individual in question and his or her environment. Assigning a medical or psychiatric diagnosis to an individual similarly can affect the individual who is thus categorized in a number of different ways. The question here is not whether the diagnosis is likely to lead to stigmatization and other negative effects or rather to a new liberating self-understanding (both positions have been argued for), but that the categorization itself *somehow* affects those who are categorized in this way.

Although all categorizations are social constructions, this means that it is important to differentiate interactive categories from other categories. Both kinds of categories are there for us to explore the meaning of, by various methods – including conceptual analysis and hermeneutical investigations. Interactive categories, however, have a special status in view of their capacity of having social effects – it is primarily the interactive kind of categories that have been made a critical focus by social constructionists.

### Theories

It is important to note that *all* scientific theories, even within the natural sciences (including quantum physics, cellular biology, and the theory of evolution) are social constructions, which have been created by researchers and acquired the status of scientific theories by gaining intersubjective recognition in the scientific community. In this capacity they can be made the object of study by researchers within the social sciences (e.g., sociologists and historians of science; e.g., Bloor 2005).

### Techniques

Techniques represent an important part of human culture. Different kinds of techniques can be found in all areas of goal-directed human activity, including house building, agriculture, industry, transport, medicine, communication, and psychotherapy. Based on the Polanyi’s (1958) writings on tacit knowledge, and Foucault (1988) on self-technologies, Lundh (2017) suggested the following general definition:

*techniques* are defined as (a) procedures designed to reach a certain goal as efficiently as possible, which (b) exist in the form of orally, textually, and/or practically transferrable knowledge, (c) that is made accessible to people by various kinds of training, education, and apprenticeship. At the psychological level this means that an individual may (d) become more or less skilled in such techniques by training and experience, and (e) thereby also acquire specific attitudes (Lundh 2017, p. 60).

According to this definition, techniques exist socio-culturally as “orally, textually, and/or practically transferrable knowledge” – that is, they represent *social-constructional* realities, products of human creativity, which are available for new generations of individuals to acquire. As this learning takes place, the individual acquires new technical skills and ways of thinking (attitudes). This psychological learning process spans over all three ontological dimensions: To learn a technique (which exists as social constructions in the form of texts or other transferrable knowledge) is to develop personal skills (which belong to the material dimension, as more or less automated behaviors and information-processing routines) and attitudes (which belong to the subjective-experiential dimension).

### A Separate Dimension, Not a Level in the Material Dimension

Finally, it is important to differentiate between the social-constructional dimension and the sociological level in Bunge’s emergent materialism, which he refers to as “social matter” (Bunge 2010, p. 84) and illustrates with sexual pairings, parenting, political fights, and work conditions. All these examples belong to the material dimension, and the same is true of such things as “democracy”, “social classes”, and “mode of production” – they belong to a sociological level in the material dimension, and not to the social-constructional dimension. In other words, sociology cannot be reduced to a study of social constructions, but also includes studies of complex human interactions that are describable in the material dimension.

## Discussion

The three-dimensional ontology that has been outlined in this paper is partly similar to Perez-Alvarez’, but it also differs in several ways. There are differences both in relation to philosophical materialism, and as to the place of psychology in a three-dimensional ontology.

### Developmental Materialism

Whereas Perez-Alvarez’ version speaks about three “genres of materiality”, the present version regards only the first dimension as material, and the other two as non-material. Still, it is seems reasonable to combine the present three-dimensional ontology with a *cosmology* where the material dimension is seen as the first dimension to develop (in the “big bang”), whereas the two other dimensions are seen as later developments. The subjective-experiential dimension may be assumed to develop in several steps during biological evolution, in connection with the development of living organisms with increasingly more complex sensory organs and nervous systems. As to the social-constructional dimension, it seems reasonable to assume that it appears later, in connection with the development of language.

Although in this view only the first dimension is material, the other two dimensions depend on the material dimension for their existence. In this sense, the present three-dimensional ontology might be referred to as a kind of developmental materialism.

### The Place of Psychology within a Three-Dimensional Ontology

The main difference between the present model and that of Perez-Alvarez concerns the place of psychology within this scheme. In Perez-Alvarez’ version, psychology is identified with the second dimension, whereas in the present version psychology spans over all three dimensions. That is, psychological science essentially involves not only the subjective-experiential dimension, but also the material dimension (in the form of situations, behaviors, brains, mental functions, information processing, etc.) and the social-constructional dimension (in the form of psychological concepts and theories which do not only aspire to describe our psychological functioning, but also affects it in various ways).

Although almost all psychological research by necessity starts from some kind of problem formulation where subjective-experiential realities play an essential role (either explicitly or implicitly), most of present-day psychological research clearly emphasizes the material dimension, and makes use of phenomenological data merely in a subordinate role. The need to be more explicit about the phenomenological starting-point for psychological research is sometimes expressed in the slogan “Back to phenomena” (e.g., Magnusson 1992).

Here it may be a relevant question to ask if psychological research may benefit from some kind of phenomenological training in the observation of experiences. Wundt and other pioneers in nineteenth century experimental psychological research used research participants who were trained in the introspective observation of their conscious experience. Although this kind of methodology fell into disrepute during the twentieth century, partly due to its basis in questionable philosophical assumptions, several groups of researchers have recently argued for new and more refined ways of training the observation of subjective experience.

Weger and Wagemann (2015), for example, advocate a rigorous form of introspection, where the observations are described in such a detailed way that they should be possible to replicate later (both by the same observer and other observers). Dorjee (2016) argues that meditation can be used to train our attention to make it into a tool not only for increased self-knowledge and personal development, but also for a deeper understanding of the mind and its place in the world, as part of a “contemplative science”. And, probably most important for the development of psychological science, Husserl’s phenomenology involves the learning of a new phenomenological attitude focused on essential characteristics of our subjective perspective. That is, unlike introspection, which is focused on *an individual’s particular experiences*, Husserl’s phenomenology is focused on *general characteristics of our subjective perspective*, which have an intersubjective status in view of the fact that we all have such a subjective perspective on the world.

The attempts to develop an empirical phenomenological psychology based entirely in the subjective-experiential dimension (e.g., Giorgi 2009), however, is not likely to succeed. In the present perspective, a mature psychological science should be able to integrate phenomenology with both natural science (e.g., neuroscience, cognitive science, behavioral science) and social constructionism. This would amount to an integration of psychological science across the material, subjective-experiential and social-constructional dimensions.

One implication of this three-dimensional ontology is that a number of fundamental questions of relevance to psychological science may be seen in a new light. For example, although the subjective perspective belongs to the subjective-experiential dimension and should not be reified as something *in* the material world, it is nevertheless the case that human beings and other animals with subjective perspectives *on* the world do exist *in* the world. We therefore need a theoretical framework that allows us to describe the level of “psycho-systems” (if Bunge’s term should be used) in a way that does justice to this essential characteristic of our way of being in the world.

One possibility is that *meaning*, seen as a core aspect of human psychological functioning, can serve as a bridging concept here. In the subjective-experiential dimension, meaning can be approached in terms such as “intentionality” (Husserl 1938/1970) and “felt meaning” (Gendlin 1962); in the social-constructional dimension it can be referred to in terms of linguistic or semantic meaning, and in the material dimension it can be approached in terms of theories of “spreading activation” in semantic networks (e.g, Collins and Loftus 1975) and meaningful aspects of the environment (e.g., “affordances”; Gibson 1979). Lundh’s (1983) theory of the human mind considered as a system of meaning structures represents a theoretical attempt to locate the phenomenon of meaning equally firmly in the subjective-experiential, the social-constructional, and the material dimensions.

## Footnotes

1 The characterization of this as not *in* the world, but as a *condition* for the experience of the world, is expressed by the term “transcendental”. The subjectivity involved is referred to by Husserl as “the transcendental ego” (as distinct from an empirical ego), and its study as “transcendental phenomenology”.

2 Analyzing the necessary conditions for something has been referred to as a “transcendental argument” or “transcendental deduction” by various philosophers such as Immanuel Kant. Husserl speaks about “transcendental logic” and “transcendental phenomenology” in this context.
